# Protective Effect of *Capparis spinosa* Extract against Potassium Bromate Induced Oxidative Stress and Genotoxicity in Mice

**DOI:** 10.1155/2021/8875238

**Published:** 2021-01-19

**Authors:** Khalid Mashai Al-Anazi, Ali Abdullah Al-Mareed, Mohammed Abul Farah, M. Ajmal Ali, Waleed A. Q. Hailan, Fahad M. Al-Hemaid

**Affiliations:** ^1^Department of Zoology, College of Science, King Saud University, P.O. Box 2455, Riyadh 11451, Saudi Arabia; ^**2**^ Department of Botany and Microbiology, College of Science, King Saud University, Riyadh 11451, Saudi Arabia

## Abstract

Despite the commercial value of potassium bromate (KBrO_3_), it has been linked to many diseases including cancer. *Capparis spinosa* possesses exceptional ethnobotanical, pharmaceutical, and economic prominence by virtue of its bioactive components. The present study was designed to explore the protective role and antioxidant potential of ethanolic leaves extract of *C. spinosa* against the oxidative stress, genotoxicity, and apoptosis induced by KBrO_3_ in an experimental animal model. The results of the study revealed remarkable diminution in the levels of oxidative stress in all the treatment groups. *C. spinosa* extract attenuated the toxic effects of KBrO_3_ significantly (*p* < 0.05) in a time- and dose-dependent manner by restoring the normal levels of ROS and antioxidative enzymes in serum and liver tissues. The extract also abolished the oxidative DNA damage as it was evident in decreased frequency of micronuclei. A marked increase in viable cells was observed in annexin-V apoptosis assay. In conclusion, the findings of the present study demonstrate that ethanolic leaves extract of *C. spinosa* has considerable protective effects against KBrO_3_-induced toxicity in experimental mice which is attributed to its antioxidant activity. Therefore, leaves of *C. spinosa* could be used as a potential source of natural antioxidant and bioactive compounds.

## 1. Introduction

Despite the commercial value of potassium bromate (KBrO_3_), in food and cosmetics industry and being a drinking water disinfection by-product, it has been and remains a paramount concern for human health [1]. KBrO_3_ has been found to induce oxidative stress in both *in vitro* and *in vivo* models [2, 3]. Recently, a new insight into oxidative stress related *in vivo* mutagenicity and genotoxicity exerted by KBrO_3_ was reported [4, 5]. Similarly, it was reported that genotoxic effects of KBrO_3_ were associated with DNA and chromosomal damage, mutations, base modification, chromosomal aberrations, and altering gene expression, leading to cancer [6–10].

Elmahdy et al. [11] stated that exposure to KBrO_3_ damaged liver tissues and also increased serum enzyme levels. In another study, KBrO_3_ was reported to induce cytotoxicity in testicular cells which were inhibited by antioxidants [12]. These findings were consistent with those obtained by Elsheikh et al. [13]. Cytotoxic effects of KBrO_3_ on kidney cells of Fischer 344 rats were also reported [14]. Stuti and D'souza [15] investigated the toxic effects of KBrO_3_ on biochemical, hematological, and histological parameters of Swiss albino mice. These findings were supported by another study that revealed that exposure to KBrO_3_ causes cell lysis in human erythrocytes [16].

*Capparis spinosa* is one of the most important economical and medicinal plants which has been used in folk medicine and considered as a very good source of glucosinolates (glucocapparin, glucoiberin, sinigrin, and glucobrassicin), flavonoids, phenolic acids, and alkaloids, all of which are known to provide health-improving benefits due to their various biological activities [17]. A wide range of biological activities such as antioxidant, anti-inflammatory, anticancer, antimicrobial, antimutagenic, and antidiabetic effects have been reported for this plant [18–22]. Furthermore, leaves of *C. spinosa* are one of the most affluent sources of quercetin which is considered as the main bio-antioxidant, hepatoprotective, and nephroprotective factor against chemically induced cytotoxicity [17, 23]. Interestingly, *C. spinosa* was found to be safe and there is negligible scientific evidence regarding any adverse or toxic effects [24, 25].

On the basis of the properties of the active compounds contained in *C. spinosa*, it is presumed that *C. spinosa* may have a potential protective role against oxidative stress, genotoxicity, and cytotoxicity in an animal model. Therefore, the present study is aimed at investigating the protective potential of *C. spinosa* ethanolic leaves extract against oxidative stress, genotoxicity, and cytotoxicity induced by KBrO_3_ in laboratory mice by applying various biochemical, cytogenetic, and cell biology methods.

## 2. Materials and Methods

### 2.1. Collection of Plant Materials and Extraction

The fresh leaves of *C. spinosa* were collected from wild habitat around Riyadh, Saudi Arabia [Voucher: FMA-MAA 27 (KSUH) (24°43′10″ N, 46°36′55″E)] and then dried and powdered. The powdered dried material (500 g leaves) was extracted with 90% ethanol, through occasional shaking and stirring till 10 days. The whole extracts were filtered and dried at room temperature. Then, the crude extract was stored at −20°C until used in the experiments.

### 2.2. Animals and Treatments

Male and female laboratory mice of the pure strain SWR/J were obtained from the Animal House, Faculty of Pharmacy, King Saud University, Riyadh. The mice were aged between 10 and 12 weeks and weighing about 28–30 g. Animals were raised and maintained in a special room at the Department of Zoology, Faculty of Science, King Saud University, under standard conditions and procedures with a temperature of 22 ± 1°C, a relative humidity of 45 ± 5%, and a light/dark cycle of 10/14 hours. The commercial food (General Corporation of Grain Silos and Flour Mills in Riyadh) and water (in clean glass bottles) were provided continuously ad libitum. Large cages (25 × 24 × 15 cm) were used for maintaining the animals, and small cages (25 × 17.5 × 14 cm) for running the experiment. All the cages were lined with an appropriate amount of clean sawdust and were washed twice a week with a water and antiseptic solution in order to prevent the growth of bacteria, fungi, and other microorganisms. The animals were divided randomly into different groups: groups treated with three sub-lethal doses of *C. spinosa* crude extract administrated intraperitoneally either alone (first phase) or simultaneously with a single sub-lethal dose (150 mg/kg bw) of KBrO_3_ [26] (second phase). Six animals were used in each group including controls.

### 2.3. Experimental Design

First phase of experiments: to evaluate the possible toxic effects and determine the safe dose of *C. spinosa* extract, three doses (100 mg/kg bw, 200 mg/kg bw, and 500 mg/kg bw) were applied for 24 h, 48 h, and 72 h duration, respectively. Subsequently, a negative control (distilled water) and a positive control (cyclophosphamide: 25 mg/kg bw) group were also maintained for comparison.

**Figure 1:**
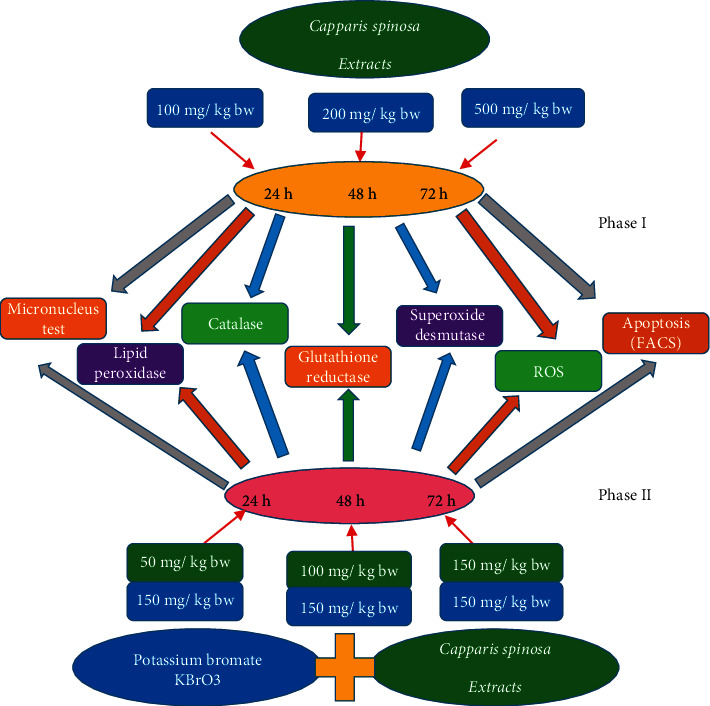
Flow chart of experimental design for the first and second phase of experiments.

Second phase of experiments: to detect the protective effect of *C. spinosa* extract, three non-toxic doses of *C. spinosa* extracts (50 mg/kg bw, 100 mg/kg bw, and 150 mg/kg bw) were applied with single sub-lethal dose of KBrO_3_ (150 mg/kg bw) simultaneously for the same durations (24h, 48 h, and 72 h). A single dose of KBrO_3_ (150 mg/kg bw) was also used alone for comparison.  Figure 1 shows the experimental design for phase I and phase II through a flow chart. All protocols followed and experiments were conducted in strict compliance with the ethical principles and guidelines of the Animal Ethics Committee, King Saud University, Riyadh, KSA.

### 2.4. Reactive Oxygen Species (ROS) Assay

A commercial kit (Image-iT™ LIVE Green Reactive Oxygen Species Detection Kit, Molecular Probes, USA) was used to assess the intracellular ROS level. Briefly, at the end of treatment durations, the animals were dissected and then liver tissue samples were obtained, homogenized in PBS, and filtered. The cell suspensions were stained with carboxy-H_2_DCFDA dye and incubated for 25 min at 37°C in the dark. For the quantitative analysis, 100 *μ*l of cell suspension was transferred in the wells of a 96-well plate and the intensity of green color was measured using a microplate reader (BioTek®, Winooski, VA, USA) at an excitation wavelength of 485 nm and an emission wavelength of 528 nm. Similarly, the qualitative analysis of ROS was achieved by fluorescence imaging with a compound microscope (Olympus BX41, Japan) fitted with appropriate fluorescence filter and a CCD camera.

### 2.5. Catalase Assay

Catalase activity assay was performed using a commercial kit (Bio-Diagnostic) by colorimetric method. Blood was obtained by cutting the tip of the tail of animal by a surgical blade. An anticoagulant such as heparin or EDTA was used to collect the blood from control and treated animals. Then, blood was centrifuged at 4000 rpm for 15 min at 4°C. The top layer of plasma was collected and used in the assay immediately or stored at −80°C. Catalase activity assay was performed by method provided in the kit. Finally, absorbance was measured of sample (*A*_Sample_) against sample blank and standard (*A*_Standard_) against standard blank at 510 nm using a Synergy microplate reader (BioTek, Winooski, VA, USA). Catalase activity was calculated by the following formula:

(1) $$\begin{array}{c} \text{catalase activity in plasma }\left(\text{U}/\text{L}\right)=\frac{\text{Abs standard}-\text{Abs sample}}{\text{Abs standard}}\times 1000. \end{array}$$

### 2.6. Reduced Glutathione Level

Reduced glutathione levels were determined using a commercial kit (Bio-Diagnostic). In brief, whole blood was collected using an anticoagulant such as heparin, or EDTA. The erythrocytes (red blood cells) were lysed in 4 times their volume of ice-cold distilled water and then centrifuged at 4000 rpm for 15 minutes at 4°C. The supernatant (erythrocyte lysate) was collected and assayed immediately or stored at −80°C. Reduced glutathione level was determined according to the method provided in the kit by reading absorbance at 405 nm and glutathione concentration in blood was determined by the following formula:

(2) $$\begin{array}{c} \text{Abs sample}\times 66\text{.}66\;\text{mg}/\text{dl}. \end{array}$$

### 2.7. Lipid Peroxidation Assay

Malondialdehyde (MDA) was measured as an indicator for a lipid peroxidation using specific kit (Bio-Diagnostic). Blood was collected without using an anticoagulant and allowed to clot for 30 min at 25°C. Then, blood was centrifuged at 4000 rpm for 15 minutes at 4°C and the top yellow serum layer was collected and either used immediately or stored at −80°C. 100 *μ*l serum for each sample was used and processed according to the method of the kit's manufacturer. Finally, the absorbance was measured at 405 nm of sample (*A*_sample_) against the blank by Synergy microplate reader (BioTek, Winooski, VA, USA).

Malondialdehyde in sample nmol/ml of serum was calculated by the following formula:

(3) $$\begin{array}{c} \text{serum}=\frac{\text{Abs sample}}{\text{Abs standard}}\times 10. \end{array}$$

### 2.8. Measurement of Superoxide Dismutase (SOD)

Liver tissue (50 mg) was cut into small pieces using a sharp blade in a porcelain apparatus with a PBS solution containing 0.16 mg/ml heparin to remove any blood cells. Tissue was homogenized in 5 ml cold buffer (100 mM potassium phosphate containing 2 mM EDTA, pH 7.0). The samples were centrifuged at 4000 rpm for 15 minutes at 4°C. The supernatant was collected and assayed immediately or stored at −80°C. Briefly, 0.5 ml of ice-cold extraction reagent was added to 1 ml supernatant in a glass test tube and vortexed for 30 seconds. Then, it was centrifuged at 4000 rpm for 10 minutes at 4°C. The aqueous upper layer was collected and assayed immediately according to the method supplied in the kit. Finally, the increase in absorbance at 560 nm for 5 min for control and sample at 25°C was recorded and SOD activity was calculated by the following formula:

(4) $$\begin{array}{c} \text{SOD activity }\text{U}/\text{gm}\text{ tissue}=\text{\% inhibition }3\text{.}75\times \frac{1}{\text{gm tissue used}}. \end{array}$$

### 2.9. Micronucleus Test (MN)

Peripheral blood was collected by cutting the tip of the tail and blood smears were made on clean microscope slides, air-dried, fixed in absolute methanol for 10 min, and stained with acridine orange (125 *μ*g/ml in pH 6.8 phosphate buffer), just before the evaluation. The slides were coded and observed in a fluorescence microscope (Olympus, BX41, Tokyo, Japan) at 40X magnification with appropriate filter settings. For the analysis of the micronucleated cells, 2000 polychromatic erythrocytes (PCE) per animal will be scored to determine the clastogenic and/or aneugenic potential of the test compound. To detect possible cytotoxic effects, the PCE/NCE (normochromatic erythrocytes) ratio in 200 erythrocytes/animal was calculated [27].

### 2.10. Annexin V-FITC Apoptosis Assay by Flow Cytometry

Annexin V- FITC apoptosis detection kit (Sigma-Aldrich, USA) was used to evaluate apoptosis. Liver cell suspensions were prepared in PBS (using cell strainer size 100 *μ*m), resuspended in 1X binding buffer, and stained with annexin V-FITC/PI for 25 min. Then, the stained cell samples were run by flow cytometry (BD FACSCalibur) and analysed by using Cell Quest Pro Software (BD).

### 2.11. Statistical Analysis

All experiments were performed in triplicate. The data were presented as mean ± S.E. A two-tailed Student's *t*-test and one-way ANOVA were applied to determine the significance at *p* < 0.05. All statistical analyses were performed using GraphPad Prism (version 7.0) software.

## 3. Results

### 3.1. Effects of *C. spinosa* Extracts (First Phase)

#### 3.1.1. Effects of *C. spinosa* Extracts on ROS Generation

**Figure 2:**
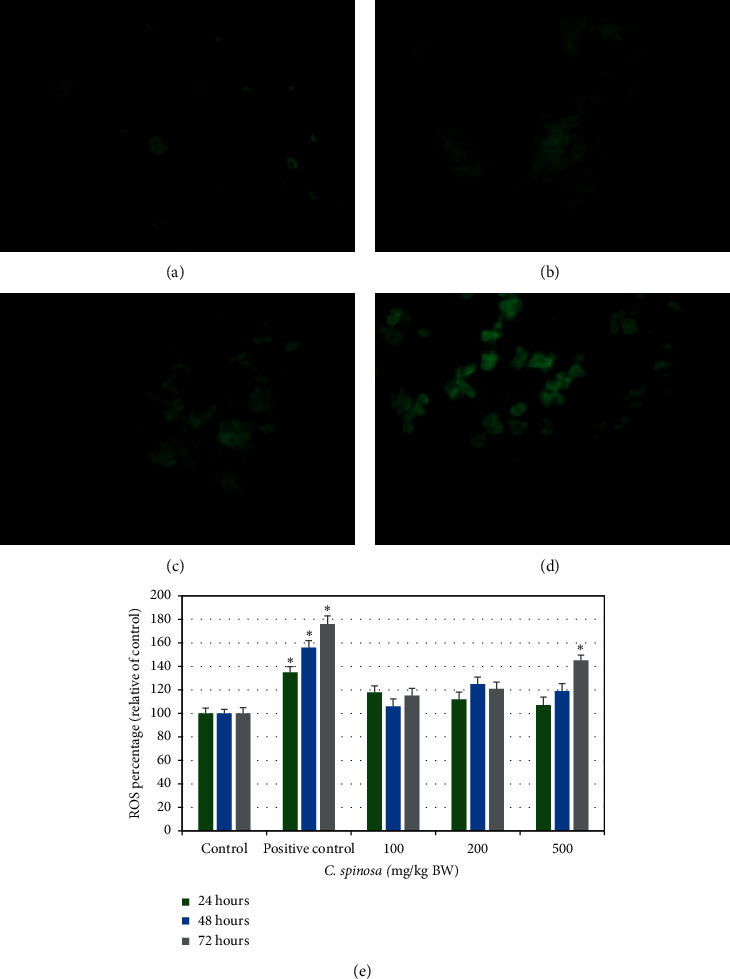
Representative images of liver cells of mice stained with carboxy-H_2_DCFDA showing effects of *C. spinosa* extracts on the generation of reactive oxygen species (ROS) treated for 72 hours. (a) Control. (b) 100 mg/kg bw. (c) 200 mg/kg bw. (d) 500 mg/kg bw. Quantitative estimation of ROS in mice treated with indicated doses for 24, 48, and 72 hours (e). Increase or decrease of ROS level is shown in percentage relative to control which is set at 100 percent. ANOVA ^*∗*^Significant (*p* < 0.05).

The fluorescence images of ROS generation in liver cells (Figures 2(a)–2(d)) show clearer elevation in the ROS level at 500 mg/kg bw dose for 72 h compared to control. However, the quantitative estimation of increase in ROS levels relative to control groups in all treatments is summarized in bar diagrams in Figure 2(e). In most of the extracts treatment groups, an increase by 10–20% was noted, whereas only in 500 mg/kg bw dose was a significant (*p* < 0.05) increase by 45% noted.

#### 3.1.2. Effects of *C. spinosa* on Oxidative Stress Parameters

**Figure 3:**
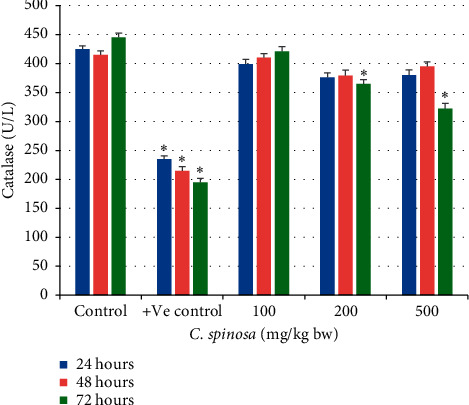
Effects of *C. spinosa* extracts on the levels of serum catalase in mice treated with indicated doses for 24, 48, and 72 hours. ANOVA ^*∗*^Significant (*p* < 0.05).

**Figure 4:**
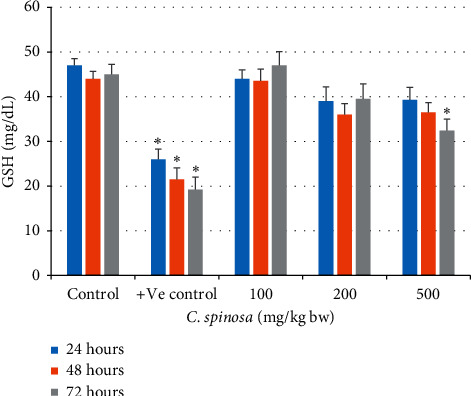
Effects of *C. spinosa* extract on the levels of reduced glutathione (GSH mg/dL) in mice treated with indicated doses for 24, 48, and 72 hours. ANOVA ^*∗*^Significant (*p* < 0.05).

**Figure 5:**
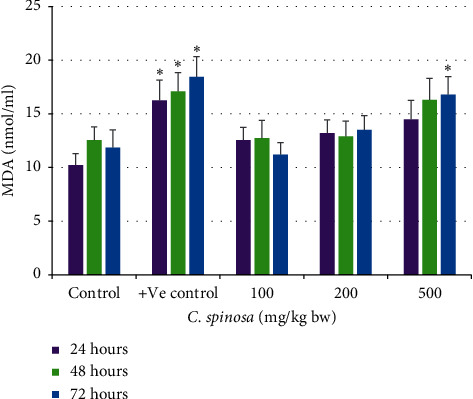
Effects of *C. spinosa* on lipid peroxidation in mice treated with indicated doses for 24, 48, and 72 hours. ANOVA ^*∗*^Significant (*p* < 0.05).

**Figure 6:**
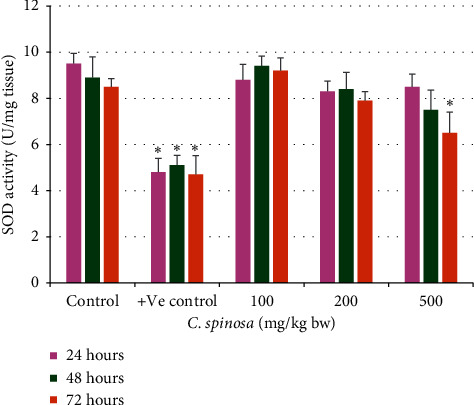
Effects of *C. spinosa* extracts on the superoxide dismutase (SOD) activity in mice treated with indicated doses for 24, 48, and 72 hours. ANOVA ^*∗*^Significant (*p* < 0.05).

The catalase activity in serum was not changed significantly in the *C. spinosa* extracts treatment group. In 72 h durations at 200 mg/kg bw and 500 mg/kg bw, catalase was significantly reduced to 365 U/L and 322 U/L as compared to control (445 U/L) as in Figure 3. At the dose 500 mg/kg bw after 72 h, the SOD activity in liver tissue lysate was also significantly decreased to 6.5 U/mg from its corresponding control (8.5 U/mg tissue). For GSH, the results in Figure 4 show that most of the treatment groups maintained similar levels of GSH as compared to control. Only at 500 mg/kg for 72 h did the GSH level drop markedly to 32.5 from control value 45 mg/dl. The similar trend of no toxicity appeared for MDA levels in serum samples of treated mice as shown in Figure 5. *C. spinosa* extracts have not much impact on the SOD activity because most of the treatments failed to deplete the SOD activity as evident from the result shown in Figure 6. In 500 mg/kg bw, after 72 h the SOD activity was significantly decreased to 6.5 U/mg tissue from its corresponding control (8.5 U/mg tissue). However, positive control (cyclophosphamide, 25 mg/kg bw) group showed significant changes in all the parameters of oxidative stress evaluated.

#### 3.1.3. Induction of Micronucleus by *C. spinosa* Extracts

**Figure 7:**
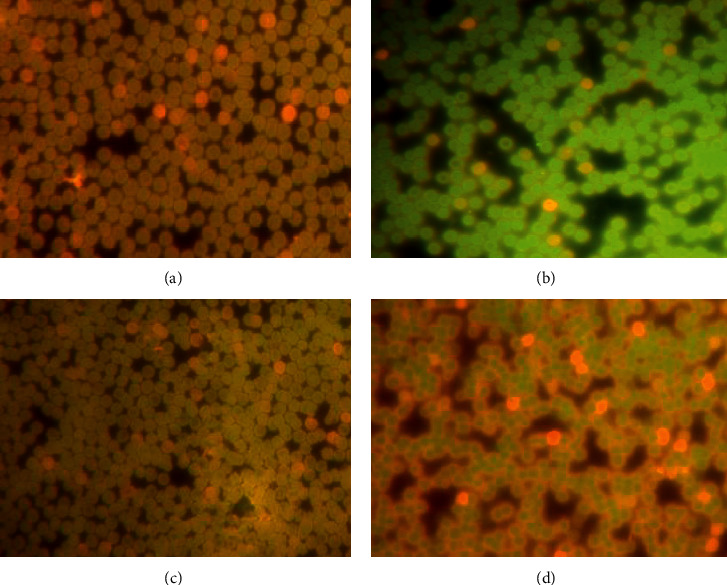
Micronucleus induction by various concentrations of *C. spinosa* extract in peripheral blood of mice after exposure for 72 hours. (a) Untreated control. (b) 100 mg/kg bw. (c) 200 mg/kg bw. (d) 500 mg/kg bw showing normal polychromatic erythrocytes (PCE orange) and normochromatic erythrocytes (NCE, green). Magnification 400X.

Table 1 represents the frequency of MN induced by *C. spinosa* extracts for all the doses applied and for three durations (24 h, 48 h, and 72 h), while Figure 7 shows the incidence of MN in extracts treatments after 72 h. However, the results show that the extract failed to induce MN as the incidence of micronuclei was insignificant (*p* < 0.05) when compared with the control group. The MN frequency in 500 mg/kg bw after 72 h exposures was found to be significant with mean MN of 5.35 ± 0.75. The remaining doses were found to register slightly higher MN frequencies compared to the control but are not significant. The minimum MN frequency was observed at 200 mg/kg bw dose after 24-hour duration which was 3.22 ± 0.51. Similarly, the ratio of PCE/NCE was also not significant in most of the treated groups. This ratio in 200 and 500 mg/kg bw was significant (*p* < 0.05) after 72 h of exposure, when compared with controls with the values of 0.96 ± 0.31 and 0.99 ± 0.35, respectively.

#### 3.1.4. Induction of Apoptosis by *C. spinosa* Extracts

**Figure 8:**
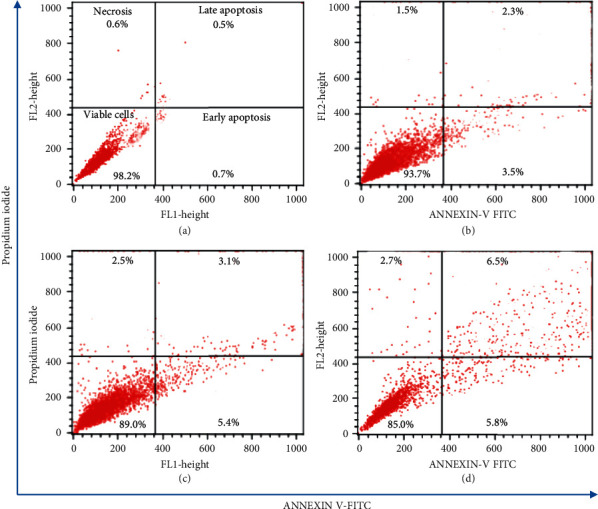
Flow cytometric analysis (annexin V-FITC/PI assay) of mice liver cells exposed to different concentrations of *C. spinosa* extracts including untreated control for 72 hours. (h) Representative dot plots showing percentage of viable cells, early apoptosis, late apoptosis, and necrotic cells. (a) Control. (b) 100 mg/kg bw. (c) 200 mg/kg bw. (d) 500 mg/kg bw.

The percentages of early and late apoptotic cells were determined by double staining with Annexin V-FITC and PI via flow cytometry. The results of apoptosis assay showed that percentage of viable cells did not change and early or late apoptotic and necrotic cells did not appear in 24 h and 48 h treatment groups in all the doses of *C. spinosa* extracts. Nevertheless, after the prolonged duration of 72 h, distinguishable enhancement in apoptotic cells and decrease in viable cells were observed in all dose groups, which are presented in Figure 8. The percentage of early apoptotic cells was recorded as 3.5%, 5.4%, and 5.8% in dose groups 100, 200, and 500 mg/kg bw, respectively, whereas late apoptosis percentages reached 2.3%, 3.2%, and 6.5% for the three doses. A maximum of 2.7% necrotic cells were also seen in 500 mg/kg bw dose. Overall, *C. spinosa* extracts were not able to induce significant apoptosis in mouse liver cells except after prolonged exposures.

### 3.2. Effects of *C. spinosa* Extracts on Toxic Impacts Induced by KBrO_3_ (Second Phase)

Preliminary screening experiments were carried out with *C. spinosa* extracts including all three treatment strategies (simultaneous, pre- and post-treatment) and simultaneous treatments were found to be more effective in ameliorating the toxic effects of KBrO_3_. Based on the findings of first phase data, three doses of *C. spinosa* extracts (50, 100, and 150 mg/kg bw) were chosen and applied simultaneously with a single dose of KBrO_3_ (150 mg/kg bw) for all three durations of 24 h, 48 h, and 72 h. The protective/antioxidant effects of *C. spinosa* extracts in combination treatment were statistically compared with the effect in KBrO_3_ (150 mg/kg bw) treatment alone.

#### 3.2.1. Effects of *C. spinosa* Extracts on the ROS Generated by KBrO_3_

**Figure 9:**
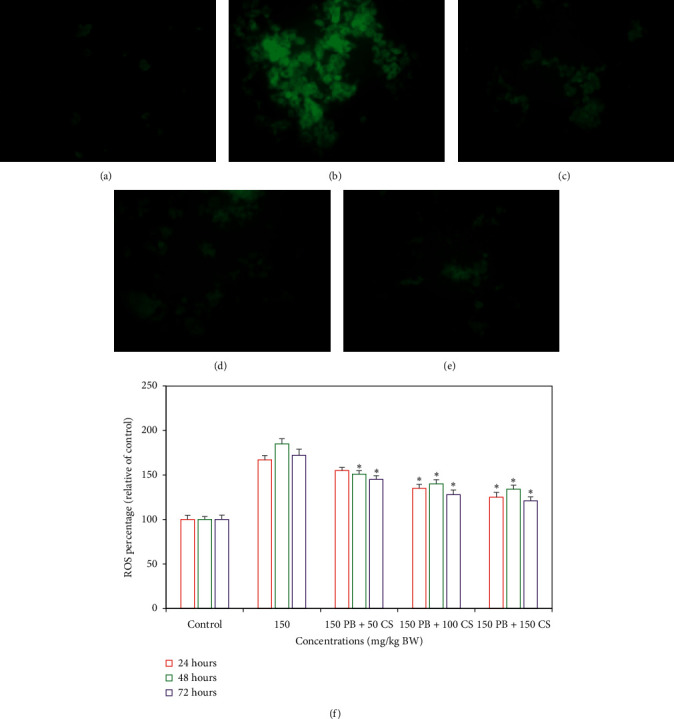
Representative images of liver cells of mice stained with carboxy-H_2_DCFDA showing effects of *C. spinosa* extracts on the generation of reactive oxygen species (ROS) induced by KBrO3 alone or in combination treated for 72 hours. (a) Control. (b) 150 mg/kg bw KBrO3. (c) 150 mg/kg bw KBrO3 + 50 mg/kg bw *C. spinosa*. (d) 150 mg/kg bw KBrO3 + 100 mg/kg bw *C. spinosa*. (e) 150 mg/kg bw KBrO3 + 150 mg/kg bw *C. spinosa.* Increase or decrease of ROS level is shown in percentage relative to control which is set at 100 percent (f). PB: potassium bromate; CS: *Capparis spinosa.* ANOVA ^*∗*^Significant (*p* < 0.05).

The representative fluorescence images for 72 h treatment are shown in Figures 9–9. Overall, the reduction in ROS after the simultaneously treatment was in a dose- and time-dependent manner. Spectrofluorometric results (Figure 9) reveal that *C. spinosa* extracts significantly (*p* < 0.05) reduced the levels of ROS in the majority of doses and all durations. The maximum percentage of 85% increase in KBrO_3_ treated liver cells was registered after 48 h. The highest reduction in ROS by *C. spinosa* extracts was detected after 72 h duration in 150 mg/kg bw dose.

#### 3.2.2. Effects of *C. spinosa* Extracts on the Catalase Level Induced by KBrO_3_

**Figure 10:**
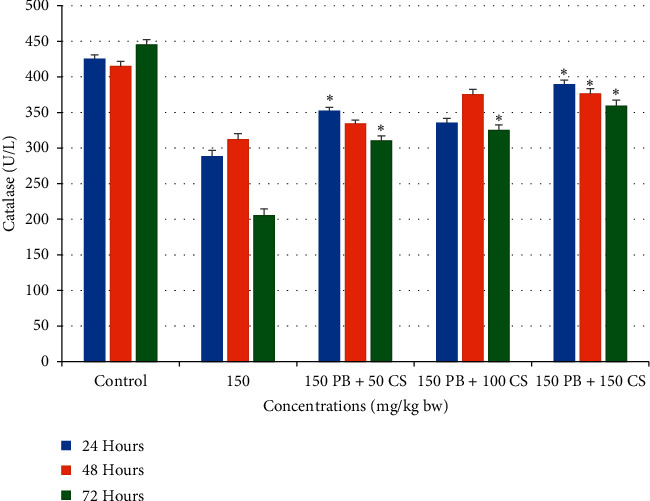
Effects of KBrO_3_ alone and in combination with *C. spinosa* extract on the levels of serum catalase in mice treated with indicated doses for 24, 48, and 72 hours. PB: potassium bromate; CS: *Capparis spinosa.* ANOVA ^*∗*^Significant (*p* < 0.05).

Effects of KBrO3 alone or in combination with *C. spinosa* extracts on the levels of serum catalase in mice treated with three doses for 24, 48, and 72 h are summarized in Figure 10. The combination treatment of *C. spinosa* extracts significantly enhanced the catalase concentration in a dose- and duration-independent manner; the outmost increase was registered at a dose of 150 mg/kg bw after 24 h which was 389 U/L after 72 h.

#### 3.2.3. Effects of *C. spinosa* Extracts on the Reduced Glutathione Level Induced by KBrO_3_

**Figure 11:**
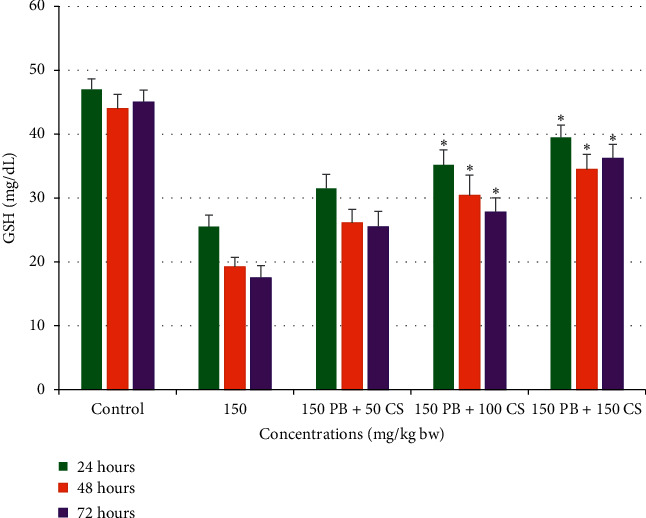
Effects of KBrO_3_ alone and in combination with *C. spinosa* extract on the levels of serum reduced glutathione (GSH mg/dL) in mice treated with indicated doses for 24, 48, and 72 hours. PB: potassium bromate; CS: *Capparis spinosa.* ANOVA ^*∗*^Significant (*p* < 0.05).

The maximum reduction in GSH level in serum of animal treated was recorded as 17.5 mg/dL after 72 h treatment of KBrO_3_ (Figure 11). Nevertheless, a significant (*p* < 0.05) increase in higher doses of *C. spinose* extracts (100 and 150 mg/kg bw) was obtained in all the durations in comparison to KBrO_3_ treated alone.

#### 3.2.4. Effects of *C. spinosa* Extracts on the Lipid Peroxidation Induced by KBrO_3_

**Figure 12:**
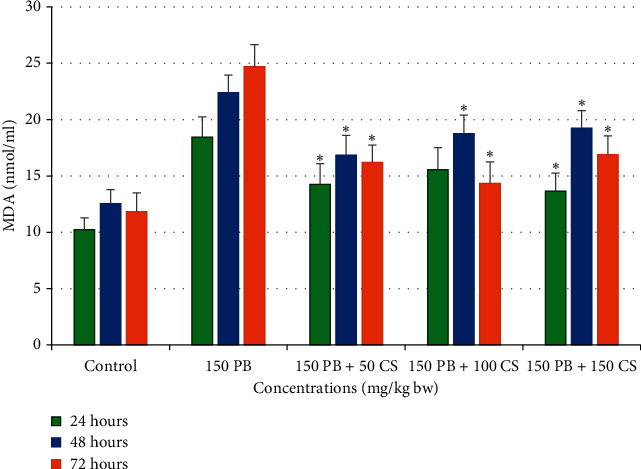
Effects of KBrO_3_ alone and in combination with *C. spinosa* extract on the levels of serum lipid peroxide (malondialdehyde, MDA) in mice treated with indicated doses for 24, 48, and 72 hours. PB: potassium bromate; CS: *Capparis spinosa.* ANOVA ^*∗*^Significant (*p* < 0.05).

As presented in bar diagrams in Figure 12, the treatment with 150 mg/kg bw KBrO_3_ alone registered about 24.7 nmol/ml MDA after 72 h duration that is about a twofold increment from control after 48 h (12.56 nmol/ml MDA). However, treatment with *C. spinosa* extracts in combination with three doses significantly (*p* < 0.05) decreased the MDA concentration in a dose- and time-independent manner.

#### 3.2.5. Effects of *C. spinosa* Extracts on the Superoxide Dismutase Activity Induced by KBrO_3_

**Figure 13:**
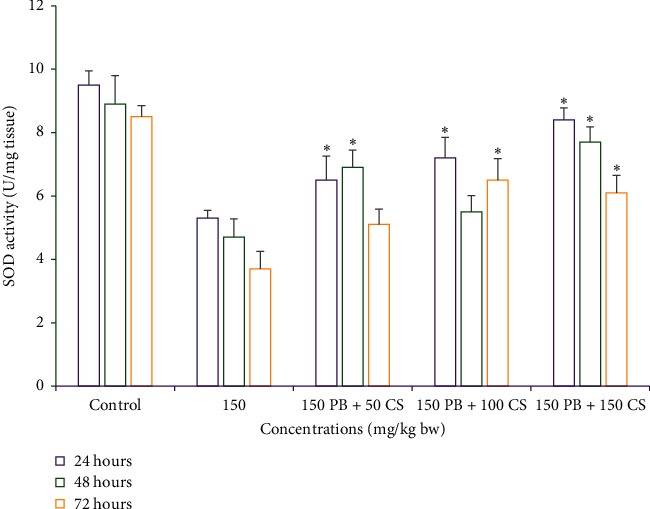
Effects of KBrO_3_ alone and in combination with *C. spinosa* extract on the levels of superoxide dismutase (SOD) activity in mice treated with indicated doses for 24, 48, and 72 hours. PB: potassium bromate; CS: *Capparis spinosa.* ANOVA ^*∗*^Significant (*p* < 0.05).

Effects of KBrO_3_ alone or in combination with *C. spinosa* extracts on (SOD) are summarized in Figure 13. KBrO_3_ (150 mg/kg bw) treated mice SOD activity was highly decreased and reached 3.7 U/mg tissue after 72 h. However, the treatment with *C. spinosa* extracts significantly (*p* < 0.05) increased the SOD activity when compared to the KBrO_3_ treated group. The highest increase in SOD activity was retained after 24 h and 150 mg/kg bw dose *C. spinose* extracts which was estimated as 8.4 U/mg tissue.

#### 3.2.6. Effects of *C. spinosa* Extracts on the Micronucleus Induced by KBrO_3_

**Figure 14:**
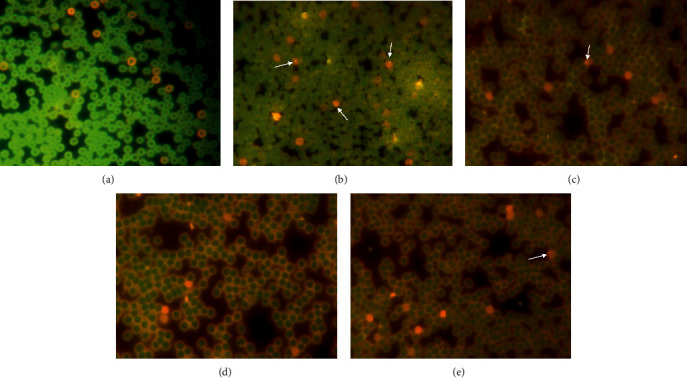
Decrease in micronucleus frequencies by simultaneous treatment by various concentrations of *C. spinosa* with KBrO_3_ in peripheral blood of mice after exposure of 72 hours. (a) Control showing normal PCE and NCE. (b) 150 mg/kg bw KBrO_3_. (c) 150 mg/kg bw KBrO_3_ + 50 mg/kg bw *C. spinosa*. (d) 150 mg/kg bw KBrO_3_ + 100 mg/kg bw *C. spinosa*. (e) 150 mg/kg bw KBrO_3_ + 150 mg/kg bw *C. spinosa* showing only few micronucleated PCE as compared to KBrO_3_ treatment alone.

The results reveal that *C. spinosa* extracts significantly (*p* < 0.05) reduced the number of micronucleated PCE in all treatments but the decrease in MN frequency was not dose-dependent except in longer duration (Figure 14, Table 2). The maximum reduction in MN was observed after 24 h where 50 mg/kg bw dose of extract recorded the MN incidence of 5.25 ± 1.35 compared to the MN frequency of 16.50 ± 1.35 in KBrO_3_ 150 mg/kg bw alone. After the treatment of 72 h, the reduction in MN frequencies in different doses of *C. spinosa* extracts in combination was observed as 13.60 ± 1.18 in 50 mg/kg bw, 12.58 ± 2.09 in 100 mg/kg bw, and 10.25 ± 2.35 in 150 mg/kg bw when compared with KBrO_3_ alone which was 21.50 ± 3.05. At 48 h, *C. spinosa* extracts decreased the MN level significantly in all the combination treatments.

#### 3.2.7. Effects of *C. spinosa* Extracts on the Apoptosis Induced by KBrO_3_

**Figure 15:**
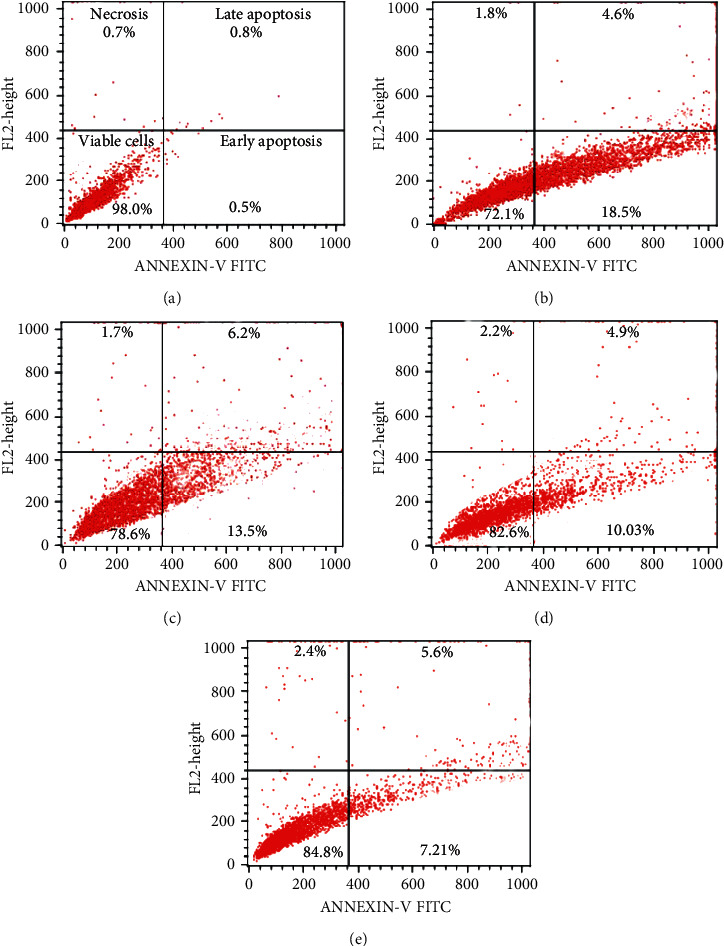
Flow cytometric analysis (annexin V-FITC/PI assay) of mice liver cells exposed to different concentrations of *C. spinosa* extracts in combination with KBrO_3_ including untreated control for 72 h. Representative dot plots showing percentage of viable cells, early apoptosis, late apoptosis, and necrotic cells. (a) Control. (b) 150 mg/kg bw KBrO_3_. (c) 150 mg/kg bw KBrO_3_ + 50 mg/kg bw *C. spinosa*. (d) 150 mg/kg bw KBrO_3_ + 100 mg/kg bw *C. spinosa*. (e) 150 mg/kg bw KBrO_3_ + 150 mg/kg bw *C. spinosa*.

Representative dot plots as presented in Figure 15 show that the percentage of early apoptotic *cells* was over 18%, while about 4.6% late apoptosis and 1.8% necrotic cells were recorded in KBrO_3_-treated liver cells. A concentration-dependent decrease in early apoptotic cells was noticed in combined treatment with *C. spinosa* extracts, which was observed as 13.5%, 10.3%, and 7.21% in 50, 100, and 150 mg/kg bw doses, respectively, after 72 h.

## 4. Discussion

*C. spinosa* has important bioactive agents that make this plant possess exceptional ethnobotanical, pharmaceutical, and economic importance [28]. Conversely, KBrO_3_ has been increasingly used as a strong oxidizing/positive agent to analyse protective effects of several natural products *in vitro* and *in vivo* [15, 29–31]. Recently, a new insight into oxidative stress related *in vivo* mutagenicity and genotoxicity exerted by KBrO_3_ was reported [4, 5]. The results of the present study demonstrated that the treatment with KBrO_3_ leads to marked elevation in ROS generation and MDA production, accompanied with GSH, catalase, and SOD depletion in the treated mice. Micronuclei formation and apoptosis induction have also been recorded due to KBrO_3_ exposure. These findings were corroborated by many reports previously [2, 10, 32].

The co-administration of *C. spinosa* leaves extract significantly ameliorated the various deleterious effects produced alone by KBrO_3_ treatment. A marked restoration of normal level of ROS and lipid peroxidation were observed in liver cells and circulating blood due to extract treatment. An increase in the activity of enzymes SOD, GSH, and catalase was also registered. The protective effects of dose 100 mg/kg of *C. spinosa* extracts were more pronounced and have ameliorated the oxidative damage with subsequent elevation in the activity level of superoxide dismutase, GSH, and catalase. So, administration of *C. spinosa* extracts alone not only did not induce any pernicious effect towards antioxidant enzymes of liver or blood, but also attenuated KBrO_3_-induced toxicity via its antioxidant effect, decreased oxidative stress, and restored the antioxidant enzymes activity. The obtained results correspond with different studies which stated that hepatoprotective effects of *C. spinosa* root bark extracts were established against CCl_4_ induced hepatotoxicity in mice [33, 34]. In the same context, Cao et al. [35] have shown that *C. spinosa* protects against oxidative stress in systemic sclerosis. A recent study revealed that hydroethanolic extracts of *C. spinosa* ameliorated cyclophosphamide induced nephrotoxicity in mice [23].

Furthermore, there were a considerable number of reports recently about the protective effects of *C. spinosa* such as antioxidant, hepatoprotective, and nephroprotective effects in various model organisms *in vivo* and *in vitro* [23, 33, 35, 36]. This is because the plants provide an alternative source to scavenge free radicals due to the presence of diverse nature of secondary metabolites [37]. The observed protective effects of *C. spinosa* might be by virtue of the high levels of polyphenolic compounds and flavonoids in the extract [36, 38].

It is known that the MN assay is a multi-end-point assay for measuring DNA damage, including the induction of micronuclei via acentric chromosome fragments and whole chromosomes, which can be distinguished by kinetochore or centromere detection using molecular methods; cytotoxicity and apoptosis are also detected [39]. It is well documented that most cytotoxic anticancer agents induce apoptosis, increasing the possibility that defects in apoptotic programs contribute to treatment failure [40]. Combined administration of *C. spinosa* extract significantly reduced the incidence of MN in peripheral blood and reduced the percentage of early and late apoptosis induced by KBrO_3_ exposure and increased the percentage of viable cells. However, the mechanism of action remains to be investigated and further studies are necessary to clarify this point. It was evident in this study that low doses of *C. spinosa* extracts were safe but high doses exerted moderate toxicity. Conversely, some of these compounds can be cytotoxic and/or genotoxic; others can be cytoprotective and/or antioxidant and antimutagenic. The obtained results justify the use of this plant since ancient time for the treatment of a lot of illnesses, such as kidney and liver disorders.

## 5. Conclusion

The current study revealed that leaves extract of *C. spinosa* containing bioactive phytochemicals exhibited remarkable antioxidant and protective potentials against the oxidative stress and toxicity induced by potassium bromate (KBrO_3_). On the other hand, *C. spinosa* extracts were lacking toxicity at low doses but they showed moderate toxicity at the highest dose (500 mg/kg bw). This was because some of the bioactive components present in crude extract might have potential toxicity at higher concentrations. Therefore, further research and development of the bioactive constituents from *C. spinosa* as potential antioxidants with possible therapeutic implications is required.

## Figures and Tables

**Table 1:** Frequency of micronucleus induced by *C. spinosa* extract in the peripheral blood of mice exposed to indicated doses for 24, 48, and 72 hours.

Treatment time (h)	Treatment (mg/kg)	MNPCE	PCE/NCE
		Mean ± SD	Mean ± SD
24	Control	3.36 ± 0.19	1.12 ± 0.25
	Positive control	9.22 ± 2.01^*∗*^	0.95 ± 0.21^*∗*^
	100	3.51 ± 0.75	1.15 ± 0.30
	200	3.22 ± 0.51	1.02 ± 0.45
	500	3.30 ± 0.32	1.11 ± 0.22

48	Control	3.22 ± 0.31	1.05 ± 0.21
	Positive control	14.22 ± 1.88^*∗*^	0.94 ± 0.23^*∗*^
	100	3.52 ± 0.65	1.16 ± 0.31
	200	3.66 ± 0.42	1.07 ± 0.35
	500	3.61 ± 0.36	1.02 ± 0.35

72	Control	3.74 ± 0.23	1.23 ± 0.19
	Positive control	17.22 ± 2.01^*∗*^	0.88 ± 0.27^*∗*^
	100	3.99 ± 0.48	1.09 ± 0.22
	200	4.25 ± 0.66	0.96 ± 0.31^*∗*^
	500	5.35 ± 0.75^*∗*^	0.99 ± 0.35^*∗*^

Student's *t*-test ^*∗*^*p* < 0.05. 2000 PCE were analysed for each animal (*n* = 5).

**Table 2:** Frequency of micronucleus induced by KBrO_3_ alone or in combination with different doses of *C. spinosa* extracts in the peripheral blood of mice exposed for 24, 48, and 72 hours.

Treatment time (h)	Treatment (mg/kg bw)	MNPCE	PCE/NCE
		Mean ± SD	Mean ± SD
24	Control	3.36 ± 0.19	1.12 ± 0.25
	150 PB	16. 50 ± 3.22	0.89 ± 0.25
	150 PB + 50 CS	5.25 ± 1.35^*∗*^	1.08 ± 0.45^*∗*^
	150 PB + 100 CS	5.75 ± 1.88^*∗*^	0.99 ± 0.34^*∗*^
	150 PB + 150 CS	9. 70 ± 2.42^*∗*^	0.98 ± 0.35

48	Control	3.22 ± 0.31	1.05 ± 0.21
	150 PB	17. 50 ± 3.22	0.85 ± 0.35
	150 PB + 50 CS	6.21 ± 1.44^*∗*^	1.04 ± 0.34^*∗*^
	150 PB + 100 CS	5.21 ± 1.48^*∗*^	0.97 ± 0.33
	150 PB + 150 CS	10.45 ± 2.05^*∗*^	0.95 ± 0.35

72	Control	3.74 ± 0.23	1.23 ± 0.19
	150 PB	21.50 ± 3.05	0.84 ± 0.31
	150 PB + 50 CS	13.60 ± 1.18^*∗*^	1.05 ± 0.39^*∗*^
	PB + 100 CS	12.58 ± 2.09^*∗*^	0.99 ± 0.42^*∗*^
	150 PB + 150 CS	10.25 ± 2.35^*∗*^	0.89 ± 0.51

Student's *t*-test ^*∗*^*p* < 0.05. PB: potassium bromate; CS: *C. spinosa* extracts. 2000 PCE were analysed for each animal (*n* = 5).

## Data Availability

The experimental results data used to support the findings of this study are included within the article. All the data could be accessed through the article after it is accepted for publication in the journal of *Evidence-Based Complementary and Alternative Medicine*. Also, the data will be available on request. There will be no restriction on data access presented within the article.

## References

[1] Ghosh P., Maity G., Banerjee S., Banerjee S. (2017). The food additive agent potassium bromate prevents growth and aggressive phenotypes by targeting multiple molecular signatures in breast cancer cells. *Experimental and Molecular Therapeutics*.

[2] Zhang Y., Jiang L., Jiang L. (2011). Possible involvement of oxidative stress in potassium bromate-induced genotoxicity in human HepG2 cells. *Chemico-Biological Interactions*.

[3] Ahmad M. K., Khan A. A., Ali S. N., Mahmood R. (2015). Chemoprotective effect of taurine on potassium bromate-induced DNA damage, DNA-protein cross-linking and oxidative stress in rat intestine. *PLoS One*.

[4] Tsuchiya T., Kijima A., Ishii Y. (2018). Mechanisms of oxidative stress-induced in vivo mutagenicity by potassium bromate and nitrofurantoin. *Journal of Toxicologic Pathology*.

[5] Obaidi I., Higgins M., Bahar B., Davis J. L., McMorrow T. (2018). Identification of the multifaceted chemopreventive activity of curcumin against the carcinogenic potential of the food additive, KBrO3. *Current Pharmaceutical Design*.

[6] Ballmaier D., Epe B. (1995). Oxidative DNA damage induced by potassium bromate under cell-free conditions and in mammalian cells. *Carcinogenesis*.

[7] Crosby L. M., Hyder K. S., DeAngelo A. B. (2000). Morphologic analysis correlates with gene expression changes in cultured F344 rat mesothelial cells. *Toxicology and Applied Pharmacology*.

[8] Murata M., Bansho Y., Inoue S. (2001). Requirement of glutathione and cysteine in guanine-specific oxidation of DNA by carcinogenic potassium bromate. *Chemical Research in Toxicology*.

[9] Spassova M. A., Miller D. J., Eastmond D. A. (2013). Dose-response analysis of bromate-induced DNA damage and mutagenicity is consistent with low-dose linear, nonthreshold processes. *Environmental and Molecular Mutagenesis*.

[10] Chauhan D., Jain P. (2016). A scientific study of genotoxic-carcinogenic impacts of Potassium Bromate as food additive on human health. *International Research Journal of Engineering and Technology*.

[11] Elmahdy M. M., Moussa M. H. G., Sh A. E., Alwazaan A. A. (2015). Pathological and Immunohistochemical study of potassium bromated on the liver of rat. *Veterinary Medicine Journal-Giza*.

[12] Nwonuma C. O., Irokanulo E. O., Iji C. E., Alejolowo O. O., Adetunji C. O. (2016). Effect of Thaumatococcus daniellii leaf rat-feed on potassium bromate induced testicular toxicity. *Asian Pacific Journal of Reproduction*.

[13] Elsheikh A. S., Fadul T. F., Aboagla E. M.-E., Rahim Gameel A. A. (2016). Effects of potassium bromate on male rat growth and testicular histology. *Asian Pacific Journal of Reproduction*.

[14] Dodd D. E., Layko D. K., Cantwell K. E., Willson G. A., Thomas R. S. (2013). Subchronic toxicity evaluation of potassium bromate in Fischer 344 rats. *Environmental Toxicology and Pharmacology*.

[15] Stuti M., D’Souza D. (2013). Effects of potassium bromate on the kidney and haematological parameters of swiss albino mice. *The Bioscan*.

[16] Ahmad M. K., Amani S., Mahmood R. (2014). Potassium bromate causes cell lysis and induces oxidative stress in human erythrocytes. *Environmental Toxicology*.

[17] Tlili N., Feriani A., Saadoui E., Nasri N., Khaldi A. (2017). *Capparis spinosa* leaves extract: source of bioantioxidants with nephroprotective and hepatoprotective effects. *Biomedicine & Pharmacotherapy*.

[18] Kulisic-Bilusic T., Schmöller I., Schnäbele K., Siracusa L., Ruberto G. (2012). The anticarcinogenic potential of essential oil and aqueous infusion from caper (*Capparis spinosa* L.). *Food Chemistry*.

[19] Nabavi S. F., Maggi F., Daglia M., Habtemariam S., Rastrelli L., Nabavi S. M. (2016). Pharmacological effects ofCapparis spinosaL. *Phytotherapy Research*.

[20] Chedraoui S., Abi-Rizk A., El-Beyrouthy M. (2017). *Capparis spinosa* L. in a systematic review: a xerophilous species of multi values and promising potentialities for agrosystems under the threat of global warming. *Frontiers in Plant Sciences*.

[21] Yu L., Yang J., Wang X., Jiang B., Sun Y., Ji Y. (2017). Antioxidant and antitumor activities of *Capparis spinosa* L. and the related mechanisms. *Oncology Reports*.

[22] Zhang H., Ma Z. (2018). Phytochemical and pharmacological properties of *Capparis spinosa* as a medicinal plant. *Nutrients*.

[23] Kalantari H., Foruozandeh H., Khodayar M. J., Siahpoosh A., Saki N., Kheradmand P. (2018). Antioxidant and hepatoprotective effects of Capparis spinosa L. fractions and Quercetin on tert-butyl hydroperoxide- induced acute liver damage in mice. *Journal of Traditional and Complementary Medicine*.

[24] Sher H., Alyemeni M. N. (2010). Ethnobotanical and pharmaceutical evaluation of *Capparis spinosa* L, validity of local folk and Unani system of medicine. *Journal of Medicinal Plant Research*.

[25] Vahid H., Rakhshandeh H., Ghorbani A. (2017). Antidiabetic properties of Capparis spinosa L. and its components. *Biomedicine & Pharmacotherapy*.

[26] Kurokawa Y., Aoki S., Matsushima Y. (1986). Dose-response studies on the carcinogenicity of potassium bromate in F344 rats after long-term oral administration. *Journal of National Cancer Institute*.

[27] OECD (2016). *Test No. 474: Mammalian Erythrocyte Micronucleus Test, OECD Guidelines for the Testing of Chemicals, Section 4*.

[28] Gadgoli C., Mishra S. H. (1999). Antihepatotoxic activity of p-methoxy benzoic acid from *Capparis spinosa*. *Journal of Ethnopharmacology*.

[29] Ke Y., Xu X., Wu S. (2013). Protective effects of extracts fromFructus rhodomyrtiagainst oxidative DNA DamageIn VitroandIn vivo. *Oxidative Medicine and Cellular Longevity*.

[30] Bayomy N. A., Soliman G. M., Abdelaziz E. Z. (2016). Effect of potassium bromate on the liver of adult male albino rat and a possible protective role of vitamin C: histological, immunohistochemical, and biochemical study. *The Anatomical Record*.

[31] Ben Saad H. B., Driss D., Jaballi I. (2018). Potassium bromate-induced changes in the adult mouse cerebellum are ameliorated by vanillin. *Biomedical and Environmental Sciences*.

[32] Ben Saad H., Driss D., Ellouz Chaabouni S. (2016). Vanillin mitigates potassium bromate-induced molecular, biochemical and histopathological changes in the kidney of adult mice. *Chemico-biological Interactions*.

[33] Aghel N., Rashidi I., Mombeini A. (2007). Hepatoprotective activity of *Capparis spinosa* root bark against CCl_4_ induced hepatic damage in mice. *Iranian Journal of Pharmaceutical Research*.

[34] Aichour R., Benzidane N., Arrar L., Charef N., Baghiani A. (2018). Hepatoprotective and anti-inflammatory activities of Algerian Capparis spinosa. L. *Annual Research & Review in Biology*.

[35] Cao Y.-l., Li X., Zheng M. (2010). *Capparis spinosa* protects against oxidative stress in systemic sclerosis dermal fibroblasts. *Archives of Dermatological Research*.

[36] Mansour R. B., Jilani I. B. H., Bouaziz M. (2016). Phenolic contents and antioxidant activity of ethanolic extract of *Capparis spinosa*. *Cytotechnology*.

[37] Jan S., Khan M. R. (2016). Protective effects of *Monotheca buxifolia* fruit on renal toxicity induced by CCl_4_ in rats. *BMC Complementary and Alternative Medicine*.

[38] Aliyazicioglu R., Eyupoglu O. E., Sahin H., Yildiz O., Baltas N. (2013). Phenolic components, antioxidant activity, and mineral analysis of *Capparis spinosa* L. *African Journal of Biotechnology*.

[39] Thomas P., Fenech M. (2011). Buccal micronucleus cytome assay. *DNA Damage Detection in Situ, Ex Vivo, and in Vivo*.

[40] Lowe S. W., Lin A. W. (2000). Apoptosis in cancer. *Carcinogenesis*.

